# Use of Race in Kidney Function Estimation: Lessons Learned and the Path Toward Health Justice

**DOI:** 10.1146/annurev-med-042921-124419

**Published:** 2023-01-27

**Authors:** Dinushika Mohottige, Opeyemi Olabisi, L. Ebony Boulware

**Affiliations:** 1 Division of Nephrology, Department of Medicine, Duke University School of Medicine, Durham, North Carolina, USA; 2 Center for Community and Population Health Improvement, Clinical and Translational Science Institute, Duke University School of Medicine, Durham, North Carolina, USA; 3 Division of General Internal Medicine, Department of Medicine, Duke University School of Medicine, Durham, North Carolina, USA; 4 Duke Molecular Physiology Institute, Duke University, Durham, North Carolina, USA

**Keywords:** equity, race-based medicine, clinical algorithms, kidney function, kidney function estimation

## Abstract

In 2020, the nephrology community formally interrogated long-standing race-based clinical algorithms used in the field, including the kidney function estimation equations. A comprehensive understanding of the history of kidney function estimation and racial essentialism is necessary to understand underpinnings of the incorporation of a Black race coefficient into prior equations. We provide a review of this history, as well as the considerations used to develop race-free equations that are a guidepost for a more equity-oriented, scientifically rigorous future for kidney function estimation and other clinical algorithms and processes in which race may be embedded as a variable.

## INTRODUCTION

Determining kidney function is essential for the accurate diagnosis, staging, and management of kidney disease, yet kidney function is challenging to precisely ascertain (1). With a global prevalence of 9.1% (nearly 700 million cases), and a US prevalence of 15% (nearly 37 million cases), chronic kidney disease (CKD) carries substantial morbidity, mortality, economic costs, and loss of quality of life for millions of individuals (2-4). Estimated glomerular filtration rate (eGFR) is a key component of assessing kidney function, and it reflects the collective filtration of plasma across all functioning nephrons in the kidneys (5, 6). In the United States, the evolution of eGFR science has been influenced significantly by the concept of race. For nearly four decades, different eGFR algorithms were used for estimating the kidney functions of Black Americans compared to White and other racial groups. Understanding the history of how the sociopolitical construct of race infiltrated the science of GFR estimation is essential to fully appreciate the history and future of kidney function estimation.

The high toll of CKD on minoritized populations is emblematic of racial, ethnic, and other inequities. The unattenuated and disproportionate morbidity and mortality from kidney disease borne by Black Americans is one of several factors prompting reconsideration of racialized GFR estimation equations (7-10). Racialized eGFR equations, which increased the approximated kidney function among Black individuals (who experience a two- to fourfold higher rate of incident end-stage kidney disease versus their White counterparts), became a central focus of discussion and calls to advance eGFR science and equity (11). In this review, we describe the historic origins of GFR estimating equations as well as factors contributing to the initial incorporation of a Black race correction factor. We then review considerations of GFR estimations with race correction, including the validity of assumptions regarding biological differences, as well as the accuracy of racial categorization, the precision of data available to inform estimations, and the generalizability and representativeness of existing equations. Finally, we review concepts around race, racism, genetics, and ancestry as well as considerations for implementing these concepts in research and future algorithms and equations.

## KIDNEY FUNCTION ESTIMATION: WHAT IS eGFR AND WHY IS IT NEEDED?

Inulin clearance (and other methodologies for directly measuring GFR, including clearance of nonradioactive iohexol and iothalamate) has long been described as a gold standard marker for estimating GFR. However, these measurements are laborious, costly, invasive, and often impractical because of the need for continuous intravenous infusion and careful timing of blood and urine collections (12). Thus, GFR is most often estimated using serum creatinine (SCr), despite its numerous limitations, including imprecision in the setting of incident and evolving acute kidney injury (13) and imprecision among individuals with atypical muscle mass [e.g., in the settings of aging, sepsis and muscle catabolism, or use of gender-affirming hormone therapies in gender-minority individuals (14-16)]. Scr is an imprecise surrogate marker for GFR because, in addition to its filtration by glomeruli, creatinine is also secreted by tubules, a process that is subject to drug interference. For instance, trimethoprim reduces tubular secretion of creatinine, thereby raising SCr level without affecting GFR. In addition, SCr is influenced by diet (e.g., high-protein diets may increase it), pregnancy, obesity, and hyperglycemia, as well as by extra renal clearance and variation in production (e.g., increased production in catabolic states, rhabdomyolysis, and severe liver disease) (15, 17). Despite these limitations, SCr measurement is widely used due to its cost-effective, rapid, and relatively noninvasive estimation of GFR.

## THE EVOLUTION OF GFR ESTIMATING EQUATIONS

Several creatinine-based GFR estimating equations exist, and the evolution of their use is relevant to current practice and the reconsideration of the race coefficient in estimation. We review several key equations below, including their defining characteristics and limitations of their use.

### Cockcroft Gault Equation

The Cockcroft Gault (CG) equation was published in 1976 in an effort to provide a rapid assessment of kidney function (18), which avoided the need for invasive and time-consuming inulin-based measurement. Prior work building on the demonstration that SCr could be related to 24-h creatinine clearance (19) laid a foundation for other investigators who aimed to predict creatinine clearance using body weight, sex (20), and age (21). The CG equation was derived using laboratory data from 249 hospitalized Canadian men of undescribed race, aged 18–92 years, to measure creatinine clearance. Actual 24-h creatinine clearance from a 24-h urine creatinine excretion test was measured against creatinine clearance calculated using four different formulas that incorporated weight, SCr, and age. However, this cohort with measured creatinine clearance ranging from 30 to 130 mL/m^2^ excluded individuals with <10 mg/kg creatinine excretion and presumably lacked race and sex diversity, though neither exclusion was explicitly discussed (18, 22). A 0.85 multiplier for women due to lower muscle mass was ultimately suggested with the goal of improving estimation, based on prior work suggesting lower SCr and urine creatinine measures in female versus male participants (20). However, the equation did not account for many non-GFR determinants of SCr (e.g., body composition, diet, medications). Despite these limitations, the ongoing use of this equation for monitoring and delivering cancer therapeutics (23) is notable. Furthermore, based on the CG formula, 95% of estimates of creatinine clearance fell within 35% of measured creatinine clearance [whereas the P_30_ for the Modification of Diet in Renal Disease (MDRD) study was 77.2, suggesting that 77.2% of GFR estimates were within 30% of measured GFR (mGFR) among patients with eGFR < 60/mL/min/1.73 m^2^] (24). However, improvements were noted in the next major equation that was developed (the MDRD equation), such that without adjustment for bias the median absolute error between measured and predicted GFR for the CG equation was 6.8 mL/min/1.73 m^2^ versus 3.8 mL/min/1.73 m^2^ using MDRD (22). The MDRD equation, which allowed for determination of eGFR (instead of creatinine clearance), was validated against ^125^I-iothalamate and did not require body weight for calculation of an estimate.

### Modification of Diet in Renal Disease Equation

After the CG equation, the MDRD equation (22), developed in 1999, was the next landmark advance in eGFR estimation because of its inclusion of Black and female participants and its utilization of mGFR, which was based on renal clearance of ^125^I-iothalamate in participants in the MDRD study. While the aim of the MDRD study was to study the effect of dietary protein restriction and strict blood pressure control on progression of kidney disease, investigators leveraged the cohort to compare creatinine-based eGFR with mGFR for the purpose of developing an equation that better estimated kidney function from SCr and other clinical factors using patients with known CKD. Using study data, investigators performed stepwise regression to develop coefficients using a training sample of 1,070 of 1,628 patients in the cohort, and subsequently applied these coefficients among a separate sample of the remaining 558 patients in the cohort to develop a GFR estimation. The initial variables considered for possible inclusion in the regression model included weight, height, sex, ethnicity, age, diagnosis of diabetes, SCr, serum urea nitrogen level, serum albumin level, serum phosphorus level, serum calcium level, mean arterial pressure, urine creatinine level, urine urea nitrogen level, urine protein level, and urine phosphorus level. A model without urine biochemical parameters was also ultimately considered because of the challenges associated with precisely measuring 24-h urine.

Although the MDRD cohort had more presumed racial and ethnic diversity than the cohort used to derive the CG equation (Table 1), many components of designation of race (termed ethnicity by the MDRD authors) were problematic in the MDRD study (22, 24). Study personnel assigned ethnicity based on skin color. Specifically, 87 Hispanic, 17 Asian, and 23 “other” individuals in the study, whose potential genetic contributions to kidney function were not considered, were considered White for the purposes of the study analysis (22).

Building on the finding that SCr values were higher in non-Hispanic Black individuals in the 1988–1994 National Health and Nutrition Examination Survey (NHANES III) study of 18,723 noninstitutionalized participants aged 12 years and older (25), the authors of the MDRD equation found that SCr levels were higher in Black adults than Whites with the same mGFR (measured by iothalamate clearance), age, and sex. While this finding was consistent with an NHANES III study by Jones et al. (25), the basis of the higher SCr among Black individuals in this study is unknown and may have been influenced by a range of factors including diet. Importantly, generalizing from these observations that all self-identified Black individuals have higher SCr than non-Black individuals was unsubstantiated. Nevertheless, Black race was included with a range (17) of other variables that were considered in the development of the MDRD GFR estimation equation. After a signal was noted in which “Black ethnicity” was independently associated with higher GFR, authors included a comment to support the use of “ethnicity,” specifically stating for corroboration, “previous studies have shown that on average, black persons have greater muscle mass than white persons” (22, p. 464). However, the cited evidence base for this comment includes anthropometric studies fraught with methodological flaws such as selection bias/convenience sampling and a lack of validated analytic tools for assessing muscle mass (11, 26-28). Ultimately, the derived four-level MDRD eGFR equation included race as a variable in addition to SCr, age, and sex, and conferred a Black race coefficient of 1.21 (29). The MDRD equation was limited by bias [median difference between mGFR and eGFR of −3.0, and interquartile range (IQR) of 21.5] and by lower precision in higher ranges of eGFR (e.g., especially above 60 mL/min/1.73 m^2^) and notably did not include many participants with diabetes, a leading cause of kidney disease in the United States.

While the original creatinine assays used to develop the original MDRD equation had substantial variability, the equation was recalibrated with standardized creatinine measures in 2006 (29). This is important because creatinine assays are subject to interference by non-creatinine moieties that react with the creatinine assay, resulting in overestimations of SCr. Despite its flaws, the MDRD equation was believed to have advanced equity because of its inclusion of more racially diverse individuals, and this equation was more accurate than the CG equation when used to estimate kidney function in a cohort of Black individuals included in the African American Study of Hypertension and Kidney Disease (AASK) (5).

### Chronic Kidney Disease Epidemiology Collaboration Equation

In 2009, the Chronic Kidney Disease Epidemiology Collaboration (CKD-EPI) equation was developed with the goal of addressing the MDRD equation’s bias and precision issues. Although the MDRD equation had advanced the field substantially compared to the CG equation, it also lacked the diversity of data inputs ultimately used to derive the CKD-EPI equation. The CKD-EPI equation was derived from a robust cross-sectional study that pooled several databases for development and validation, all of which used different (30) exogenous markers [e.g., iothalamate, iohexol in several European studies, EDTA (ethylenediaminetetraacetic acid)] to measure GFR. In total, data from 9,254 individuals with and without CKD from 10 studies were used for the development of the equation, which was validated in 3,896 individuals in 16 different cohorts. The eGFRs were then compared to population prevalence estimates of CKD in NHANES. Multiple regression was used with inclusion of race as a predictor variable. Stepwise multivariable regression was used to determine variables that predict GFR, which included creatinine level, sex, race, and age. Internal validation involved comparing predicted GFR to mGFR, and external validation involved the same assessment along with comparison of predicted GFR to other markers. Ultimately, a Black race correction factor of 1.159 was included in the equation, based on the observation that mGFR among Black individuals in the cohort was on average 15.9% higher than among non-Black individuals with the same creatinine level, sex, and age. The authors also stated that age, race, and sex are surrogates for non-GFR determinants of creatinine level and associated with muscle mass, the main determinant of creatinine generation. Ultimately, authors concluded that this CKD-EPI equation should replace the MDRD equation, and multiple studies have supported its improved accuracy in estimating GFR, especially at higher ranges (17, 31-33).

However, the CKD-EPI equation has several notable limitations. First, it built on the MDRD study, retaining its a priori hypothesis of Black race as a predictor of kidney function/SCr and using the MDRD cohort in the development data sets. Second, it is notable that the cohorts that contribute a large percentage of Black individuals are from the Chronic Renal Insufficiency Cohort (CRIC) study (43% Black, 289 participants) and AASK (100% Black, 1,807 participants). As other authors have highlighted, the lack of diabetic individuals in AASK, which included self-identified Black/African Americans with hypertension and eGFR 20–65 mL/min/1.73 m^2^ raises questions regarding the representativeness of this cohort (11), as does the fact that nearly 50% of this cohort had an annual income below $15,000. Further complicating the racial categorization in the CKD-EPI equation development is the overall underrepresentation of Asian, Hispanic, Native American, Pacific Islander, and other racial and ethnic categorizations. Recently, investigators have noted the importance of including genetic ancestry in the determination of eGFR (to avoid racial categorizations) (34). However, the genetic ancestry estimations often conducted have focused on developing an admixture model using individuals from projects like the 1000 Genomes Project as a reference. Ultimately, many of these studies have relied on conceptualizing genetic ancestry into racial categories that mirror US Census racial category designations (e.g., African, European) without providing precise data on genetic risk factors implicated in excess kidney disease risk (35-38). In these equations, a racial (or “ethnic”) variable defined dichotomously as “black” versus “white and other” was applied across study participants, who are classified across four poorly defined (32) and inconsistently determined (22) categories, “Black,” “Hispanic,” “Asian,” and “White or other,” within their respective study cohorts. Notably, the resulting widely used two-race CKD-EPI equation was created using validation cohorts inclusive of populations in the United States, Canada, and Europe (Netherlands, Sweden, France, Denmark) (32) without mention of how race and ethnicity are sociopolitically structured, dynamic, and variably defined by participants and/or others (e.g., research and clinical staff, government agencies like the US Census) across these contexts within the United States and in other nations (e.g., in France, where a proportion of “Black” participants in the validation cohort were recruited) (39, 40). More specifically, it is unclear what it means to be self- or otherwise-identified as “Black” in a French versus US context, or even within a US context. Finally, muscle mass, diet, and medications that interfere with tubular secretion were not accounted for in any models.

### Combined Creatinine–Cystatin C Equation

In an effort to avoid reliance on creatinine-based GFR estimation tools, investigators began exploring the use of cystatin C in an equation to estimate kidney function (41). Cystatin C had been demonstrated to be less influenced by diet (42) and possibly more predictive of adverse outcomes than creatinine, especially among older adults (43). Investigators developed two new equations, one with cystatin C alone and one combining it with creatinine, using a development data set including 5,352 individuals across 13 studies. Linear regression was used to relate mGFR to creatinine, cystatin C, age, and sex, and additional variables included Black race, diabetes status, and weight (44). Notably, when these equations were compared to the performance of the creatinine-based GFR measures and prior equations developed for standardized cystatin C values (41), the combined creatinine–cystatin C equation performed better than equations based on each marker alone and was stated to be a useful confirmatory test for CKD (44, 45). Although cystatin C had been previously noted to be a more accurate test for eGFR among individuals with low muscle mass and less subject to the effects of age, sex, and race, investigators demonstrated that the use of an eGFR equation based solely on cystatin C was not more accurate than creatinine-based measures (44). While providing a substantial advance in the field, the CKD-EPI creatinine–cystatin C equation continued to provide a Black correction factor, though the CKD-EPI cystatin C equation did not.

## GLOBAL CONSIDERATIONS OF GFR ESTIMATING EQUATIONS USING A RACE CORRECTION

An important follow-up study to CKD-EPI equations aimed to expand their global application, yet unveiled the challenges associated with their conceptualization of race/ethnicity and the incorporation of a Black race coefficient. Authors aimed to create a more globally applicable kidney function estimation equation with a four-level (versus two-level) race variable, and thus take into account differences in other racial and ethnic groups, specifically Native American and Hispanic and Asian individuals, for whom study authors “hypothesized that the performance of the CKD EPI equation could be improved” (46, p. 556). This involved incorporating the original CKD-EPI development cohorts and a subsequent validation of a four-level equation using cohorts from the United States, Canada, and Europe, as well as from studies conducted in China, Japan, and South Africa. Authors noted that CKD-EPI could be used in its two-variable form for all racial/ethnic groups in the United States and Europe, with the caveat that GFR estimates may vary in accuracy among and within racial and ethnic groups. The two-level and four-level race equations result in substantial bias nearing 15 mL/min for Black individuals with eGFR > 90 mL/min/1.73 m^2^ and significant bias nearing 30–35 mL/min in Japanese and South African cohorts. These findings would confer substantial changes to kidney disease management (46). Furthermore, the CKD-EPI and MDRD equations in their original forms demonstrated substantial bias in Japan (47, 48), China (49), South Africa (50, 51), Ghana (52), Pakistan (53), and India (54), findings that have been corroborated in numerous additional studies from across the globe, including in nations with substantial racial and ethnic admixture (e.g., Brazil) (55, 56) and Singapore (57). This finding of poorer performance of the equation with a Black coefficient in African cohorts is especially notable given its contradiction of the faulty “racial essentialism” premise upon which the Black race modifier is built. In short, the poorer performance negated the hypothesis that individuals racialized as Black or with African ancestry/origins would be collectively defined by a unique characteristic, in this case eGFR. Poor performance of CKD-EPI equations with the Black race coefficient was also demonstrated among two cohorts of individuals residing in Europe with “African” ancestry (58, 59), in which the Black race coefficient overestimated kidney function substantially.

## CONTEXTUALIZING THE USE OF RACE IN GFR ESTIMATING EQUATIONS: RACE AND RACIAL ESSENTIALISM

To appreciate the controversy associated with the use of race in these equations, it is important to review the concepts of race and racial essentialism—and the risks these concepts confer in the context of using eGFR to guide medical decision making.

### Race

Race is not a genetic/biological risk factor for poor kidney health outcomes or muscle mass; nor is it an appropriate surrogate for SCr (11, 60). However, race is a potent risk factor for racism and other inequitable experiences in racialized societies, including the United States. In March 2021, the American Society of Nephrology (ASN) and National Kidney Foundation (NKF) proclaimed that “race is a social, not a biological, construct” (61, p. 1) counteracting decades of historic and current medical research and practice in which race has been erroneously conflated with a fixed biological and/or genetic essence and with diseases, such as sickle cell anemia (62). At its conception, race was promoted as a key feature within a taxonomy whereby individuals in our human species were hierarchically categorized into phenotypically defined (e.g., by skin color, hair texture) racial groupings. Race was subsequently used as a sociopolitical tool through which policies, practices, and ideologies founded upon the inferiority of non-White, and especially Black, individuals were explicitly and implicitly embedded into scientific discourse and medical pedagogy by Enlightenment-era scientists (8, 63-65). For centuries, race has been a basis for scientific inquiry, medical pedagogy, abuse (e.g., eugenics), a range of clinical algorithms in which the “otherness” of Black individuals is embedded into clinical algorithms including the interpretation of pulmonary function tests (63), and differential treatment of Black and other minoritized individuals.

The incorporation of a Black race correction factor into eGFR equations illuminates how perceptions of racial difference can be embedded within widely disseminated practices touted as empirical and objective, despite their failure to (*a*) assess and account for true biological disease risk factors (7, 11) or (*b*) appropriately define race and ethnicity within the sociopolitical and historic context of lived experience (i.e., racism, poverty, marginalization) (66, 67). The implementation of this correction factor further did not elucidate associations or mechanisms through which any structurally mediated factors (e.g., interpersonal racism, exposure to harmful environmental toxins and other adversity) may become embodied over time in kidney health (68, 69). Although ancestry has been proposed as a potential way to increase the specificity of racial categories (34, 70), evidence suggests this approach is flawed due to imprecision in ancestry measurement (71-73) and inconsistent correlations of ancestry with socially assigned race (73-78).

### Racial Essentialism

Racial essentialism may be defined as the view that racial groups possess an underlying essence or “fixed, natural, uniform and defining characteristics” that represent informative unalterable traits including genetics, phenotypic features, behaviors, and ability (79, p. 3), which define group members. This essentialism undergirds a historical desire to use social markers like race to classify patterns in phenotypes (e.g., higher muscle mass) and conditions, to the detriment of scientific inquiry. It has validated and reinforced the use of race (and ethnicity) as a basis for presupposing biological differences in health without any solid scientific basis, including the development of kidney function estimating equations (80, 81). Specifically, this essentialism may have contributed to the hypothesis that a nonobjective Black race (rather than other unmeasured factors) predicted higher creatinine in the cohorts used to develop the MDRD and CKD-EPI equations (63, 82). Moving forward with further efforts to improve precision in clinical algorithms and predictive equations, racial essentialism should be recognized as a threat to rigorous science seeking to understand validated biological mechanisms contributing to individual health differences.

## IMPLICATIONS OF RACIALIZED eGFR EQUATIONS

A groundswell of concerns regarding the use of race in equations and the role of racial essentialism in driving its use led investigators to better understand the potential harms to Black individuals caused by the racialized eGFR equation. In 2019, multiple US organizations began to reconsider the use of the race coefficient in prior equations, including MDRD and CKD-EPI 2009. Over a 10-month period between September 2020 and June 2021, the ASN and NKF convened a multidisciplinary task force of stakeholders, including patients and a range of experts, to carefully identify 26 approaches for estimating GFR that did and did not consider race. They evaluated each approach based on assay availability and standardization, implementation, population diversity for equation development, performance compared to mGFR, consequences to clinical care population tracking and research, and patient centeredness (5, 6). This process of deliberation also involved gathering oral testimonies from patients, providers, trainees, and community members, who evaluated an interim report (5), as well as influential studies that emerged demonstrating impact of removal of the race coefficient.

Concerns were raised regarding nonstandardized categorization of race and the implementation of the Black race coefficient in populations that self-identify as multiracial. There were also concerns regarding appropriate dosing of medications, access to kidney transplantation, nephrology care, and appropriate CKD management (7, 26). Proponents of maintaining the race coefficient cited concerns that eliminating the coefficient might limit the potential inclusion of Black individuals in clinical trials, possibly lead to underdosing chemotherapeutic agents, and potentially decrease Black individuals’ access to medications including metformin, for which use of an eGFR threshold for eligibility was noted to possibly decrease sex- and race-based disparities in prescribing (versus use of creatinine-based thresholds) (83-85). Proponents of eliminating the coefficient cited the potential harms of maintaining it, particularly because of the unique disadvantages conferred to individuals racialized as Black in the United States, who bear a staggering and disproportionate burden of morbidity and mortality from end-stage kidney disease. These disparities are pronounced and include persistently inequitable access to high-quality CKD care (86), appropriate kidney replacement therapies (2, 87, 88), and kidney transplantation among Black individuals (89, 90).

In a study from a single healthcare system (91), investigators discovered that removal of the Black race coefficient from CKD-EPI substantially increased the estimated CKD prevalence among Black individuals. Separately, a study of US non-veteran Black individuals and Black veterans found that an additional 981,038 Black non-veterans overall and nearly 84,988 Black veterans would be classified as having CKD in the United States (92) if the race coefficient were removed. Similar findings were demonstrated based on hypothetical removal of the coefficient from the MDRD equation (93, 94). Diao et al. (95) conducted a similar analysis examining the potential effect of removing the Black race coefficient, based on NHANES data from 2001 to 2018. They estimated that one million additional Black adults would have a new CKD diagnosis (estimated crude prevalence 14.9% to 19.4%), which would have substantial implications for key kidney health-promoting interventions including eligibility for Medicare coverage for nutrition therapy, eligibility for nephrology referral, and kidney transplant waitlisting, as well as eligibility for key kidney risk-reducing therapies like SGLT2 inhibitors (9, 95). Further work in transplantation confirmed the harms of the racialized eGFR equations in exacerbating existing transplant inequities, including waitlisting (96, 97). For instance, Zelnick et al. (98) investigated the accuracy of the racialized CKD-EPI equation and its potential contribution to kidney transplant inequities for Black individuals in three complementary analyses conducted among 1,658 self-identified Black individuals enrolled in the CRIC study. Removal of the race coefficient resulted in Black individuals reaching an eGFR of 20 mL/min/1.73 m^2^ or less (thus reaching the transplant waitlisting threshold 1.9 years sooner) (98). Furthermore, in this analysis, Black individuals had a 52% higher risk of achieving an eGFR < 30 mL/min/1.73 m^2^, which is the threshold suggested by the Organ Procurement and Transplantation Network for initiating discussions about kidney replacement options including living donation.

After extensive deliberation, a consensus agreement by the ASN and NKF affirmed the importance of ensuring accurate CKD surveillance (99) while avoiding reinforcing the erroneous notion that so-called race and ethnicity capture static objective biological and behavioral facts about difference, rather than politically, socially, geographically, and historically patterned, hierarchical, and dynamic constructs (63, 100). Recommendations of the joint ASN-NKF task force include immediate implementation of the CKD-EPI (2021) creatinine-based equation without the race coefficient (101) in all laboratories because of its diverse development cohort and acceptable performance characteristics. The committee also recommended national efforts to increase use of cystatin C, particularly in cases with clinical ambiguity about the accuracy of using creatinine alone.

In light of mounting evidence about widening racial inequities perpetuated by continued use of racialized kidney function estimation equations, especially among Black Americans, as well as the recent 2022 unified ASN-NKF task force recommendations to move toward a race-free equation for estimating GFR (6, 101), it is critical that investigators approach CKD surveillance with a nuanced lens that recognizes the role of (*a*) unique genetic risk factors to assess kidney function, (*b*) novel biomarkers that can provide adequate prediction of end-stage kidney disease and staging, and (*c*) cost-effective approaches including assessment of albuminuria for ensuring that CKD surveillance and kidney disease management is a global priority (9, 91, 95).

## THE FUTURE OF eGFR ESTIMATION AND RACIALIZED ALGORITHMS

Reassessing the validity of race-based medicine, such as questioning the use of a coefficient that made kidney function better among the group that suffered worse outcomes, fueled reconsideration of racialized algorithms for kidney function estimation. This process required a continuous and iterative process of reckoning with the systematic embedding of race, racial essentialist ideologies, and racialized medical practices in healthcare and other key institutions and systems. Moving forward, clarity, transparency, and patient-centered conversations about the prior equations and the future of nonracialized algorithms are needed, along with concerted efforts to dismantle other key contributors to racial inequities. This change must include a careful understanding of the history and origins of these algorithms, as well as caution as investigators consider novel biomarkers, race, and ancestry in future predictive equations. We also recommend the active pursuit of precision and contextualization in risk prediction models, including in the terms and variables used as proxies to explain differences in kidney function across defined populations. Finally, humble and multidisciplinary engagement is needed to reform inscribed falsehoods around race and biology (64). This will involve implementing race-conscious antiracist frameworks like those proposed by Cerdena et al. within scientific studies that engage rigorous genetic science, so that we can better elucidate how social and genetic factors may interact to manifest as differential kidney disease risk and outcomes (63).

Lessons learned from the reckoning with race-based kidney function estimation have renewed hope for a more equity-oriented, scientifically rigorous future. The people and communities we serve may be harmed by the inappropriate use of race and ethnicity to make sense of population-level and genetic differences in health, without a clear or accurate understanding of their role in reinforcing health inequities and egregious power and resource imbalances. As scientists, clinicians, and public health professionals, we must pursue the precision required to deliver global health equity in kidney disease surveillance and treatment.

## Figures and Tables

**Table 1:** eGFR estimation equations, included populations and considerations of race and correction factors

Equation	Population	Definitions of ethnicity or race	Method	Correction factors and considerations	Equation
Cockcroft Gault (18)	249 veterans aged 18–92, no mention of underlying kidney disease	Ethnicity and race are not mentioned, but the study was conducted in Canada	Determined CrCl using two 24-h measurements of Cr excretion, which were then compared to prior normogram and formulas which incorporated SCr, and age	Added no race correction but did add a sex correction factor based on prior work Did not use standardized Cr measures	CrCl (male) = ([140 – age] × weight in kg)/(SCr × 72) CrCl (female) = CrCl (male) × 0.85
MDRD 6-item and standardized 4-item equation	1,628 overall patients mean mGFR 40 mL/min/1.73 m^2^, primarily glomerular disease or unknown etiology of CKD *n* = 983 Male (60%) *n* = 187 Black (12%) *n* = 1,304 White included 87 individuals designated as Hispanic, 17 Asian, and 23 “other”race Included only 34 (2%) individuals with eGFR >90 mL/min/1.73 m^2^ and only 156 (9.6%) with eGFR <15 mL/min/1.73 m^2^	Ethnicity was assigned by study personnel, without explicit criteria, probably by examination of skin color	Urinary clearance of iothalamate	Added race coefficient and determined eGFR instead of CrCl	MDRD demographic and serum variables only GFR = 170 × [Pcr]^−0.999^ × [age]^−0.176^ × [0.762 if patient is female] × [1.180 if patient is Black] × [SUN]^−0.170^ × [Alb]^0.318^ Re-expressed 4-variable MDRD (GFR 175 × standardized Scr^−1.154^ × age^−0.203^ × 1.212 [if Black] × 0.742 [if female]) (29)
CKD-EPI	*n* = 8,254 total; *n* = 5,504 development cohort, *n* = 2,750 in internal validation cohort, *n* = 3,896 in external validation cohort Includes patients with diabetes, hypertension, and kidney transplant Mean mGFR = 68 mL/min/1.73 m^2^ in development cohort *n* = 1,728 (32%) Black in development cohort, *n* = 857 (31%) Black in internal validation cohort, *n* = 384 (10%) in external validation cohort Key contributors to “Black” participants are AASK, MDRD, and CRIC participants Other racial categories include Hispanic and Asian. White and other are categorized together	Specifics of race group ascertainment across studies including European cohorts are denoted in supplemental tables available in the original reference (101). Note that in European cohorts, there is no clear ability to designate similar racial categories as used in the US context. There are no consistent definitions for race across each cohort used for the development or validation data sets	Urine clearance of iothalamate for development data set	Compared to MDRD, has less bias (median difference between mGFR and eGFR is 2.5 versus 5.5), improved precision (IQR of differences 16.6 versus 18.3), and greater accuracy (% eGFR within 30% of mGFR is 84.1% versus 80.6% for MDRD)	GFR = 141 × min(S_cr_/κ, 1)^α^ × max(S_cr_/κ, 1)^−1.209^ × 0.993^age^ × 1.018 [if female] × 1.159 [if African American], where S_cr_ is serum creatinine in μmol/L, κ is 61.9 for females and 79.6 for males, α is −0.329 for females and −0.411 for males, min indicates the minimum of S_cr_/κ or 1, and max indicates the maximum of S_cr_/κ or 1
CKD-EPI cystatin C equation and CKD-EPI combined Cr–cystatin C equation	Development and internal validation *n* = 5,352 *n* = 2,123 (40%) Black race in internal validation data set *n* = 30 (3%) Black race in external validation data set In the development data set, the mean (±SD) mGFR was 68±39 mL/min/1.73 m^2^	Specifics of race group ascertainment across studies including European cohorts are noted supplemental tables available in the original reference (101) Note that in European cohorts, there is no clear ability to designate similar racial categories as used in the US context. There are no consistent definitions for race across each cohort used for the development or validation data sets	Urine clearance of iothalamate Also used standardized measures of Cr and cystatin C	KDIGO 2012 recommends this combined equation as a confirmatory test Combined equation performs better in terms of precision (IQR of difference 13.4 versus 15.4 for Cr alone or 16.4 for cystatin C alone) and also has more accuracy (8.5% estimates versus 12.8% for Cr alone and 14.1% for cystatin C alone) CKD-EPI cystatin C equation does not include a Black race coefficient, as this was not found to improve its performance The combination equation utilizing Cr and cystatin C was notably most precise and accurate for estimating GFR across a range of GFR even among individuals with BMI < 20	The CKD-EPI cystatin C equation (2012) can be expressed as a single equation: 133 × min (SCys/0.8, 1)^−0.499^ × max (SCys/0.8, 1)^−1.328^ × 0.996^age^ [× 0.932 if female], where min indicates the minimum of SCr/κ or 1, and max indicates the maximum of SCys/κ The CKD-EPI Cr–cystatin C equation (2012) can be expressed as a single equation: 135 × min (SCr/κ, 1)^α^ × max (SCr/κ, 1)^−0.601^ × min (SCys/0.8, 1)^−0.375^ × max (SCys/0.8, 1)^−0.711^ × 0.995^age^ [× 0.969 if female] [× 1.08 if Black], where κ is 0.7 for females and 0.9 for males, α is −0.248 for females and −0.207 for males, min indicates the minimum of SCr/κ or 1, and max indicates the maximum of SCr/κ or 1

Abbreviations: AASK, African American Study of Hypertension and Kidney Disease; Alb, albumin; BMI, body mass index; CKD, chronic kidney disease; CKD-EPI, Chronic Kidney Disease Epidemiology Collaboration; Cr, creatinine; CrCl, creatinine clearance; CRIC, Chronic Renal Insufficiency Cohort; eGFR, estimated glomerular filtration rate; GFR, glomerular filtration rate; IQR, interquartile range; KDIGO 2012, Kidney Disease Improving Global Outcomes 2012; MDRD, Modification of Diet in Renal Disease; mGFR, measured glomerular filtration rate; SCr, serum creatinine; SCys, serum cystatin C; SUN, serum urea nitrogen.

## Abbreviations

P_30_: the percentage of patients with estimated glomerular filtration rate within +30% of measured glomerular filtration rate
